# Validation of a network-based strategy for the optimization of combinatorial target selection in breast cancer therapy: siRNA knockdown of network targets in MDA-MB-231 cells as an *in vitro* model for inhibition of tumor development

**DOI:** 10.18632/oncotarget.11055

**Published:** 2016-08-04

**Authors:** Tatiana M. Tilli, Nicolas Carels, Jack A. Tuszynski, Manijeh Pasdar

**Affiliations:** ^1^ Laboratory of Biological System Modeling, National Institute for Science and Technology on Innovation in Neglected Diseases (INCT/IDN), Center for Technological Development in Health (CDTS), Oswaldo Cruz Foundation (Fiocruz), Rio de Janeiro, Brazil; ^2^ Department of Oncology, Faculty of Medicine & Dentistry, University of Alberta, Edmonton, Alberta, Canada; ^3^ Department of Physics, University of Alberta, Edmonton, Alberta, Canada

**Keywords:** cancer therapy, network-based strategy, siRNA therapy, triple-negative breast cancer (TNBC)

## Abstract

Network-based strategies provided by systems biology are attractive tools for cancer therapy. Modulation of cancer networks by anticancer drugs may alter the response of malignant cells and/or drive network re-organization into the inhibition of cancer progression. Previously, using systems biology approach and cancer signaling networks, we identified top-5 highly expressed and connected proteins (HSP90AB1, CSNK2B, TK1, YWHAB and VIM) in the invasive MDA-MB-231 breast cancer cell line. Here, we have knocked down the expression of these proteins, individually or together using siRNAs. The transfected cell lines were assessed for *in vitro* cell growth, colony formation, migration and invasion relative to control transfected MDA-MB-231, the non-invasive MCF-7 breast carcinoma cell line and the non-tumoral mammary epithelial cell line MCF-10A. The knockdown of the top-5 upregulated connectivity hubs successfully inhibited the *in vitro* proliferation, colony formation, anchorage independence, migration and invasion in MDA-MB-231 cells; with minimal effects in the control transfected MDA-MB-231 cells or MCF-7 and MCF-10A cells. The *in vitro* validation of bioinformatics predictions regarding optimized multi-target selection for therapy suggests that protein expression levels together with protein-protein interaction network analysis may provide an optimized combinatorial target selection for a highly effective anti-metastatic precision therapy in triple-negative breast cancer. This approach increases the ability to identify not only druggable hubs as essential targets for cancer survival, but also interactions most susceptible to synergistic drug action. The data provided in this report constitute a preliminary step toward the personalized clinical application of our strategy to optimize the therapeutic use of anti-cancer drugs.

## INTRODUCTION

Breast cancer (BC) accounted for approximately 1.67 million new cancer cases in 2012 (25% of all cancers) worldwide with an estimated number of 522,000 deaths [1]. Although significant improvements in diagnosis and treatment have been made in the past few years, BC remains the third leading cause of death globally and its mortality rate has not changed significantly since 1992. Sadly, because of drug resistance, the vast majority of BC patients experience tumor progression and relapse following the first round of chemotherapy. However, due to recent advances in medical research, tumors can now be classified according to molecular targets, which allows personalized treatment with drugs that specifically inhibit these targets to improve clinical outcomes [2, 3]. Precision medicine requires the assessment of a substantial number of molecular combinations resulting from intra-tumoral heterogeneity and human haplotype variability that underlie the malignant progression [4–7]. The shortcomings of *one-size-fits-all* treatments are well reflected in the often disappointing outcomes of current chemotherapies, where drugs directed at an individual target frequently show limited efficacy and safety due to factors such as off-target interactions, bypass mechanisms and cross-talk across compensatory escape pathways [8].

One of the major hallmarks of cancer is dysregulation of gene expression in malignant cells [9]. Recent progress in high-throughput generation of transcriptome, proteome, and interactome data together with the *in silico* data mining offers a new and promising opportunity to identify key protein targets that are of marginal implications in normal cells, but represent molecular signaling hubs in cancer cells [10–15]. Ample body of evidence has shown that an efficacious cancer treatment requires multi-drug therapeutics [16]. The question is which of the hundreds of available compounds should be selected for personalized treatment and what would be the optimized combination therapy composed of in order to maximize efficacy and minimize potential side effects.

The use of systems biology approaches to address cancer research has been recently proposed both as a conceptual organizing principle and a practical tool for therapy selection [17]. It has been recently demonstrated that the probability of 5-year patient survival [18] is inversely proportional to the complexity of the signaling network [17, 19] for the types of cancer considered in this study.

In order to design a strategy of protein target identification that would allow the development of therapeutic strategies with the lowest level of deleterious side effects possible, we compared the gene expression pattern of different malignant cell lines representative of the main forms of breast cancer by subtracting their gene expression level (RNA-seq) from those of a non-tumoral cell line used as a reference. The genes found to be upregulated in malignant cell lines by comparison to the reference were considered potential targets for drug development because the transient inhibition of their expression should not affect the living condition of the reference cells. Among the 150-300 upregulated genes in malignant cells, some have a larger likelihood of being suitable targets for drug development than the others because they warrant a larger protein connectivity rate in the cell-line-specific sub-networks induced by signaling rewiring during the oncogenesis process [20]. To rank the likelihood of potential protein target according to the benefit of their inhibition to patients by a precision therapy, we used degree-entropy as a measure of protein connectivity. Proteins acting as connectivity hubs in the signaling network of malignant cell lines were found by comparing transcriptome (RNA-seq) to interactome data. Normalized RNA-seq data allow the inference of the signaling proteins that are effectively expressed in a given malignant cell line by comparison to non-tumoral cell line used as a reference. The local degree-entropy associated to each expressed proteins can be calculated from the interactome data and used to rank the relative connectivity rate according to the total degree-entropy associated to the whole network as well as to rank the comparative benefits of drug cocktails to patients according to the profile of their upregulated top connectivity hubs [21, 22]. These analyses identified a network of 5 genes: HSP90AB1 (a member of the heat shock family of proteins), CSNK2B, (casein kinase 2β), TK1 (thymidine kinase 1), YWHAB (a member of the 14-3-3 family of proteins), and VIM (vimentin, a type III mesenchymal intermediate filament) that have also been reported to be upregulated in breast cancer [23–31]. In the present study, we validate the five upregulated most connected *hubs* (top-5) in the protein interactome of MDA-MB-231 as specific targets for potential therapeutic application in precision medicine of cancer by their knockdown using interfering RNA (siRNA) [17, 20–22]. We show that the inactivation of these 5 targets in MDA-MB-231 cells significantly decreases cell proliferation, colony formation, anchorage-independent cell growth, cell migration and cell invasion. This proof-of-concept study can serve as a preliminary step in the process of drug discovery towards development of precision therapies.

## RESULTS

### Protein levels and key target knockdown

**Figure 1:**
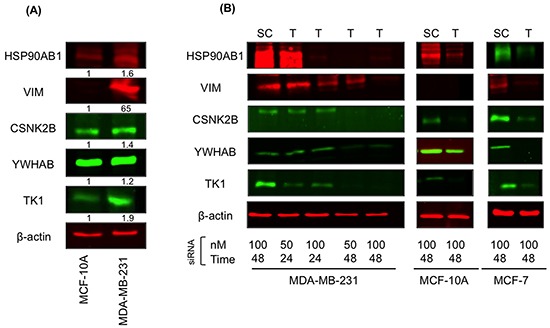
Network target proteins are overexpressed in MDA-MB-231 cells **A.** Total cellular proteins from MDA-MB-231 and MCF-10A cells were processed for immunoblotting with HSP90AB1, CSNK2B, TK1, YWHAB, and VIM antibodies as described in Materials and Methods. **B.** MDA-MB-231, MCF-10A and MCF-7 cells were transfected with scrambled siRNAs or siRNA targeting HSP90AB1, CSNK2B, TK1, YWHAB, and VIM at 50 and 100 nM concentrations for 24 and 48 h. Total cell lysates from transfectants were processed for immunoblotting as described in (A). The same blots were probed with β-actin antibodies to confirm equal loading. Results are representative of 3 independent experiments. SC, scrambled siRNA; T, Network target siRNA.

**Figure 2:**
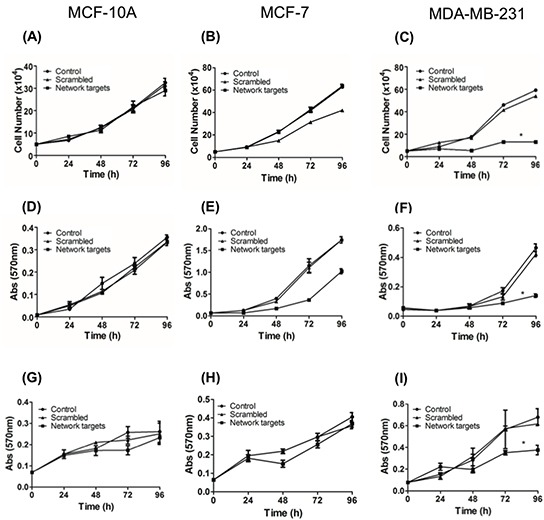
The knockdown of the network targets components inhibits the MDA-MB-231 proliferation Triplicate cultures of MCF-10A (A,D,G), MCF-7 (B,E,H) and MDA-MB-231 (C,F,I) cells remained untransfected or transfected with the scrambled or network targets siRNAs and processed for growth and survival assays. Cultures were trypsinized at 24, 48, 72 and 96 h and the number of viable cells assessed following trypan blue staining **(A-C)** or crystal violet staining **(D-F)** or MTT assay **(G-I)** as described in Materials and Methods. Differences in growth and survival rates among various cell lines and treatments were determined using ANOVA. *p < 0.0001. The lack of error bars is due to small differences among various cell lines.

**Figure 3:**
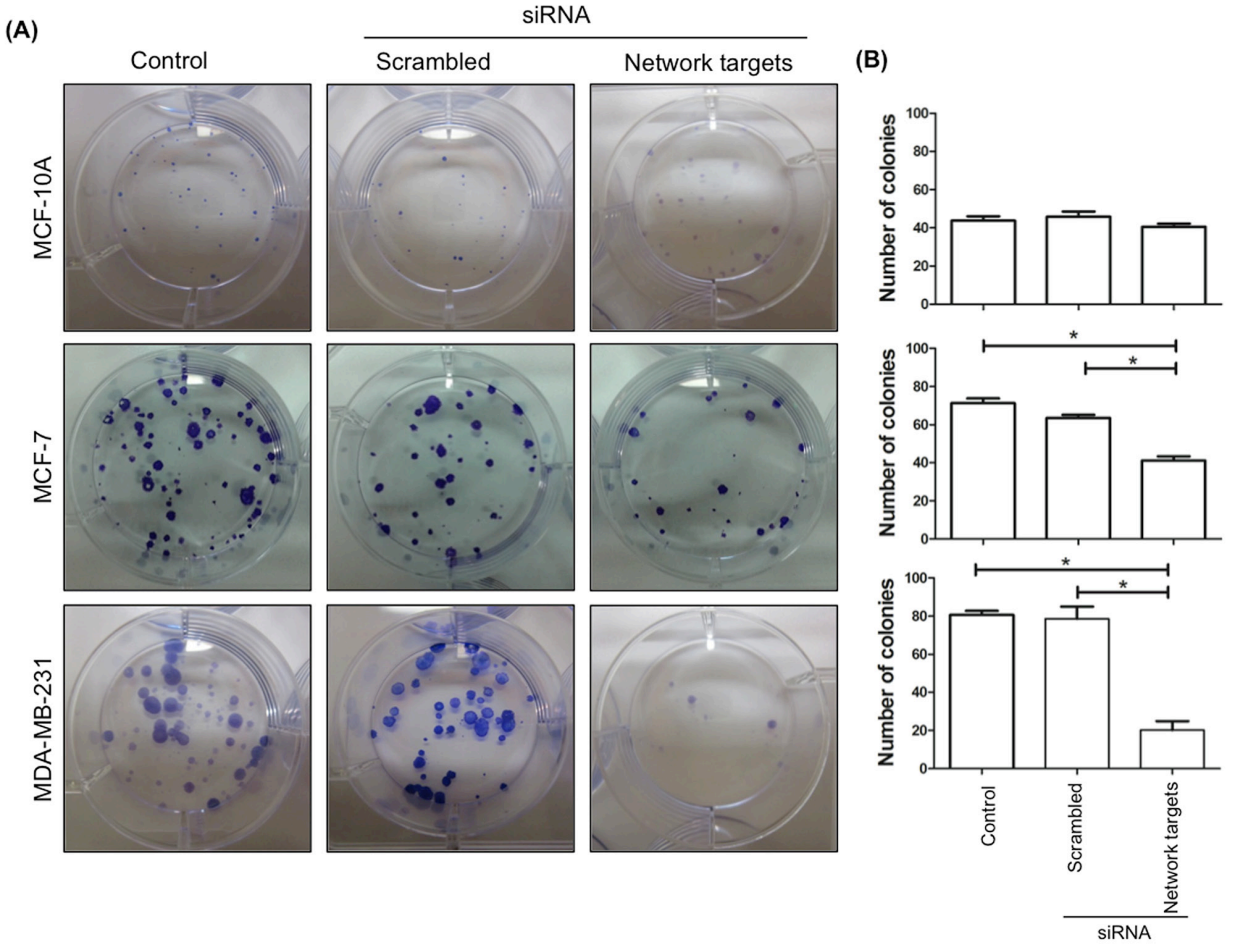
The knockdown of the network targets inhibits focus formation in MDA-MB-231 cells **A.** MCF-10A, MCF-7 and MDA-MB-231 cells remained untransfected or transfected with the scrambled or network targets siRNAs. Cells were plated 48 h post transfection at 5 × 10^2^ cells/well in 6-well plates and foci formation was detected by crystal violet staining after 15 days. **B.** Quantitation of the assays in (A). Colonies were counted for all cell lines and histograms represent the average of 3 experiments for each cell line for each treatment. Statistically significant differences were determined by the Dunnett's test. *p < 0.0001

**Figure 4:**
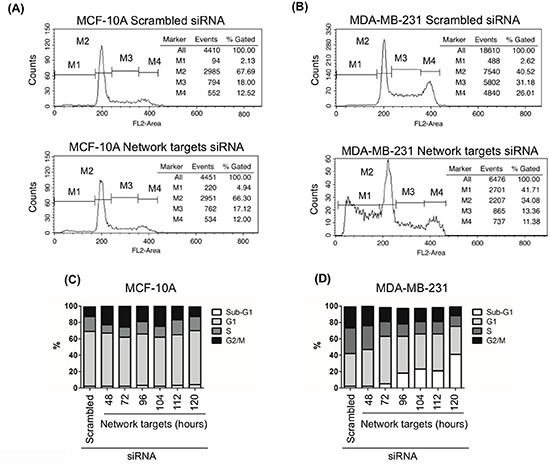
The knockdown of the network target induces cell death in MDA-MB-231 cells MCF-10A and MDA-MB-231 cells were plated at 90% confluence. Cells were transfected with scrambled or network targets siRNAs. At 48, 72, 96, 104, 112 and 120 h post transfection cells were stained with propidium iodide and analyzed by flow cytometry. The percent of dead cells are indicated by M1 in **A-B.** and by sub-G1 in **C-D.** Cell cycle phases were examined 48 h after transfection (A-B).

**Figure 5:**
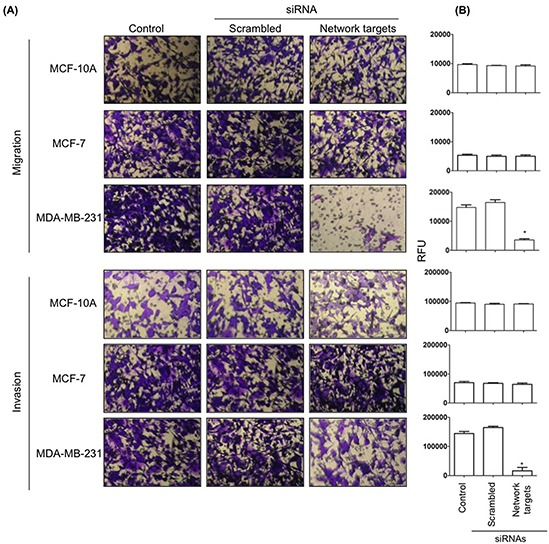
The knockdown of the network targets decreases cell migration and invasion in MDA-MB-231 cells MDA-MB-231, MCF-10A and MCF-7 remained untransfected or were transfected with scrambled or network targets siRNAs and processed for 24 h *in vitro* migration and invasion assays as described in Materials and Methods. Migrating and invading cells were stained with crystal violet and photographed using a 20X objective of an inverted microscope **A.** The number of migrated/invaded cells was quantified using Calcein-AM according to the manufacturer's protocol as described in Materials and Methods. Histograms represent the average ± SD of 3 independent experiments. *p < 0.0001.

**Figure 6:**
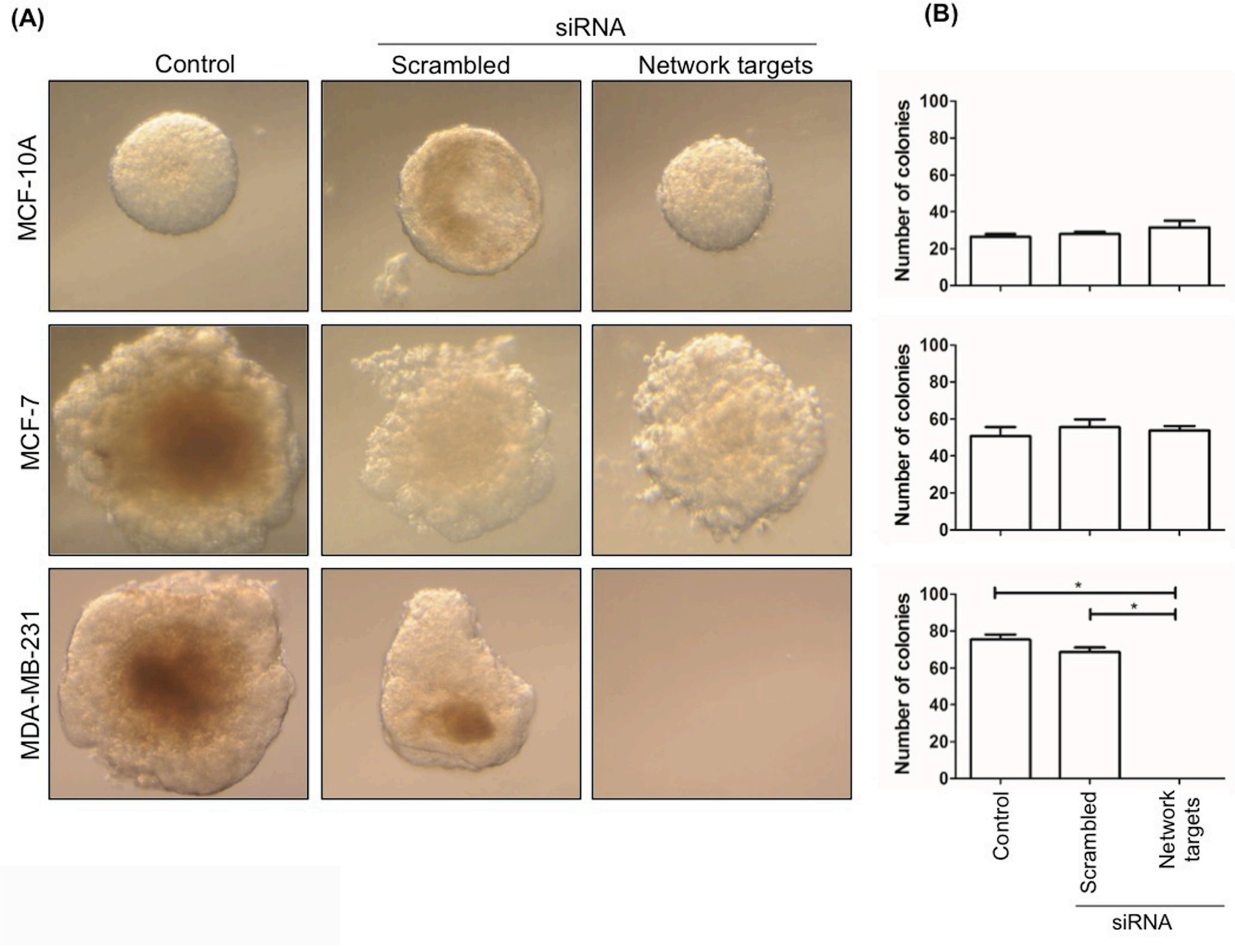
The knockdown of the network targets decreases the growth of MDA-MB-231 cells in soft agar MDA-MB-231, MCF-10A and MCF-7 remained untransfected or were transfected with scrambled or network targets siRNAs. Cells were inoculated onto soft agar to assess their ability to grow without support. Plates were examined 4 weeks after inoculation **A.** The histograms in **B.** represent the average number of colonies ± SD of 3 independent experiments. Statistically significant differences were determined by Dunnett's test. *p < 0.0001.

**Figure 7:**
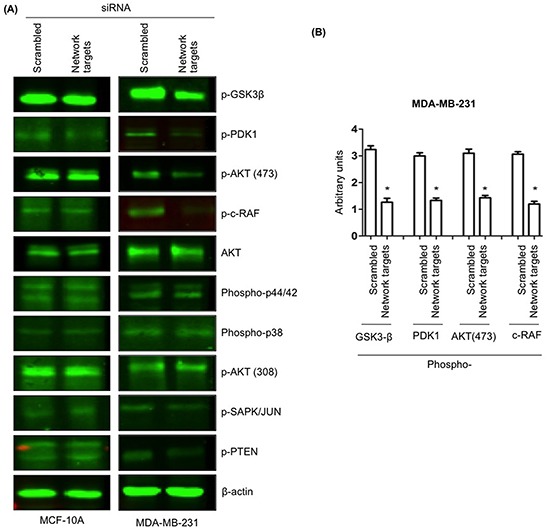
The knockdown of the network targets downregulates phosphorylation of PI3K/Akt and MAPK signaling pathways in MDA-MB-231 cells **A.** MCF-10A and MDA-MB-231 cells were transfected with scrambled or network targets siRNAs. Forty-eight hours after transfections, total cell lysates from various cultures were processed for immunoblotting with pGSK-3β, pPDK1, AKT, pAKT (473 and 308), p-c-RAF, Phospho-p44/42, Phospho-p38, p-SAPK/JUN, pPTEN and β-actin antibodies. **B.** Quantitation of the decreased pGSK3β, PDK1, AKT (473) and c-RAF in MDA-MB-231 transfected with network targets siRNA cells. *p < 0.05.

We validated the results of our *in silico* analysis by processing lysates from MDA-MB-231 and MCF-10A cells for immunoblotting with HSP90AB1, CSNK2B, TK1, YWHAB, VIM and β-actin (control) antibodies. The results showed increased protein levels for all targets except YWHAB in MDA-MB-231 relative to MCF-10A cells (Figure 1A). To assess the role of the network targets in the *in vitro* tumorigenic and metastatic behavior of MDA-MB-231 cells, all 5 targets were knocked down in MDA-MB-231, MCF-7 and MCF-10A cells. All cell lines were transfected with two different concentrations (50 and 100 nM) of siRNA combination (HSP90AB1, CSNK2B, TK1, YWHAB, VIM). The downregulation of the corresponding proteins was assessed after 24 and 48 h using immunoblotting of cell lysates with the respective antibodies. All proteins were effectively downregulated (>90%) in MDA-MB-231 and MCF-7 cells. In MCF-10A cells, HSP90AB1, CSNK2B, TK1, VIM were also reduced by >90% whereas YWHAB was decreased by ~60% (Figure 1B). We then proceeded with functional assays using individual (Supplementary Figures S1 and S2) as well as combined (Figures 2–7) network targets knockdown transfectants. Knockdown of individual member of the network targets had no observable effect on anchorage-independent/cell growth, survival, foci formation, or cell migration and invasion. In contrast, the knockdown of the combined network targets had profound effects on *in vitro* growth, migratory and invasive properties of MDA-MB-231 cells suggesting a synergistic rather than an additive effect of multiple siRNA transfections on malignant signal network.

### The expression of the network targets is essential to MDA-MB-231 cell growth and survival

Increased cell growth is one of the main cellular events associated with tumor development and progression. Thus, we asked whether the knockdown of top-5 network targets modifies the growth behavior of the breast cell lines under the study. To address this question, single cell replicates of MCF-10A, MCF-7, and MDA-MB-231 cells remained untransfected or transfected with scrambled siRNA or target siRNAs. The trypsinized cells counted at 24, 48, 72 and 96 h showed no significant growth differences among untransfected, scrambled or target siRNAs transfected MCF-10A cells (Figure 2A). In MCF-7 cells, untransfected and scrambled siRNA transfected cultures showed similar growth patterns whereas cells transfected with target siRNAs grew slower although not significantly (Figure 2B). In contrast, the growth of target siRNA transfected MDA-MB-231 cells was significantly lower (*p* <0.0001) than the untransfected or scrambled siRNA transfected cultures (Figure 2C).

To further analyze this difference in growth capacity, we performed a growth kinetics assay accompanied by crystal violet incorporation, which correlates with total cell numbers. Consistent with the previous assay, we noted a significant reduction in the growth of MDA-MB-231 cultures treated with target siRNAs (Figure 2F) whereas there were no differences in growth kinetics of MCF-10A and MCF-7 cells (Figure 2D, E).

The survival of various cell lines after transfection with scrambled and target siRNAs was also evaluated by an MTT assay (see Materials and Methods). This assay detected no difference between untransfected cells and scrambled or target siRNAs transfected MCF-10A cells (Figure 2G). MCF-7 cells transfected with the target siRNA also showed a decrease in survival relative to the untransfected and scrambled siRNA transfected cells although not significantly different (Figure 2H). The survival of MDA-MB-231 cells transfected with the target siRNAs was significantly lower (*p* <0.0001) than the untransfected and scrambled siRNA transfected cells (Figure 2I).

We further assessed changes in foci formation upon network targets knockdown. Focus-forming assays were performed as described in Materials and Methods. At the end of 2-weeks in culture, a few small foci were detected in untransfected as well as scrambled siRNA and target siRNA transfected MCF-10A (Figure 3A, B, Top). In contrast, we detected a ~40% reduction in the number of foci in MCF-7 cells transfected with the target siRNAs relative to the control and scrambled siRNA transfected cells (Figure 3A, B, Middle). Knocking down network targets was much more effective in reducing foci formation in MDA-MB-231 cells, which showed 75% reduction in the number of foci (Figure 3A, B, Bottom).

### Knockdown of the key network targets induces cell death in MDA-MB-231

To assess the effect of network targets knockdown on cell death, we measured changes in the sub-G1 DNA content as described in Materials and Methods. In MCF-10A cells, ~5% of the cells transfected with target siRNA exhibited sub-G1 DNA content (Figure 4A, C). A similar rate of cell death was found in MDA-MB-231 cells transfected with scrambled or target siRNAs for 48 h (Figure 4B, D). However, the number of cells in sub-G1 in this cell line increased with longer Post-transfection times. By 120 h post-transfection, ~40% of MDA-MB-231 cells had sub-G1 DNA content, indicating inhibition of cell cycle progression and massive induction of cell death (Figure 4B, D).

### Knockdown of the key network targets inhibits MDA-MB-231 cell migration and invasion

*In vitro* migration and invasion assays were performed and quantitated as described in Materials and Methods. There were no changes in the migratory and invasive properties of MCF-10A and MCF-7 cells transfected with network targets siRNAs (Figure 5, Migration, Invasion; A, B). In contrast, there was a significant reduction in both migration and invasion of MDA-MB-231 cells transfected with target siRNAs. When compared to untransfected or scrambled siRNA transfected cells, MDA-MB-231 network targets knockdowns showed a 70% and 90% decrease in migration and invasion, respectively (Figure 5, Migration; Invasion, A, B, MDA-MB-231).

The results of the migration and invasion assays confirmed the biological significance of the *in silico* selected network targets [20] in MDA-MB-231 and led us to further examine the effect of their knockdown on the anchorage-independent growth of these cells as a measure of their metastatic potential [32]. We found that the knockdown of the top-5 network targets had very little or no effect on the anchorage-independent growth of MCF-10A or MCF-7 cells. In contrast, MDA-MB-231 cells transfected with network targets siRNAs were completely unable to grow (Figure 6A, 6B).

The results of the functional assays collectively suggest that the network targets genes may act as tumor/metastasis promoters and play a major role in the regulation of metastatic potential.

### Knockdown of the key network targets and its consequences for MDA-MB-231 cell signaling

During tumor progression, tumor cells undergo molecular alterations that lead to the anchorage independent/uncontrolled growth and inhibition of cell death as well as acquisition of migration and invasion potential [33]. Ideally, an effective drug combination should target signaling pathways that are involved in the above-mentioned processes. The activation of phosphatidylinositol 3-kinase (PI3K-Akt) and mitogen-activated protein kinase (MAPK) signaling pathways is well documented to promote both tumor development and progression [33]. To this end, we examined if the knockdown of key network targets genes would modulate PI3K-Akt and MAPK activity. Forty-eight hours after the transfection of MCF-10A and MDA-MB-231 cells with the scrambled or network targets siRNAs, cell extracts from all transfectants were processed for immunoblotting with phospho-specific antibodies to detect various activated signaling molecules as depicted in Figure 7. The analysis of the immunoblots from MCF-10A lysates showed no detectable differences between lysates prepared from scrambled or target siRNA transfected cells (Figure 7, MCF-10A). In contrast, a ~1.5-fold decrease in phosphorylation levels of Akt (Ser473), GSK3β, PDK1 and c-Raf was detected in MDA-MB-231 cells transfected with target siRNAs relative to the scrambled siRNA transfectants (Figure 7A, B, MDA-MB-231).

## DISCUSSION

We have validated the prediction that the top-5 upregulated connectivity hubs identified based on bioinformatics inferences in a protein-protein interaction network analysis are potentially suitable targets for the development of drug combinations against cancer [20, 21].

The overexpression of the top-5 genes, inferred from RNA-seq analysis [20], in MDA-MB-231 cells compared to the reference MCF-10A cells was validated by immunoblot analysis. Here, MCF-10A was used as a reference since its immortal transformation allows its *in vitro* culture and it is not malignant. Functional assays demonstrated that the overexpression of the 5 most connected network targets significantly contribute to the proliferative and invasive phenotype of MDA-MB-231. Consistent with the bioinformatics inferences [20], the knockdown of the network targets had little or no effects on growth, migration and invasion of the non-invasive MCF-7 or reference MCF-10A cells.

The components of the PI3K/Akt and MAPK pathways, including GSK3β, PDK1, and c-RAF have been reported to be involved in breast cancer progression. GSK3β is a versatile enzyme involved in numerous signaling pathways that influence metabolism, embryogenesis, differentiation, migration, cell cycle progression, and survival; including those activated by growth factors, Wnts, cytokines, hedgehog and G protein-coupled ligands [34, 35]. Recent data indicate that alteration of PDK1 (Phosphoinositide-dependent kinase 1) contributes to the oncogenic PI3K signaling in breast cancer progression [36]. Dupuy et al. [37] showed that PDK1 is required for efficient formation of liver metastases. RAF is a component of the MAPK/ERK signaling pathway that contributes to tumor initiation, progression and metastasis [38]. MAPK/PI3K cross-talk regulates Rac/Cdc42/PAK signaling and cytoskeletal reorganization [39–41]. In the present study, we have observed that downregulation of signaling network targets significantly decreased the phosphorylation of Akt (473), GSK3β, PDK1, and c-RAF in MDA-MB-231. These observations explain the reduction of cell proliferation, survival, migration and invasion that we observed upon network targets knockdown. Although the participation of key canonical pathways, such as those involving PI3K-Akt, MAPK, IGF, FGF, MET and mTOR [2], is well established in different cancers, these pathways cannot capture the complex and context-dependent cellular rewiring patterns behind distinct cancer phenotypes. For instance, many genes identified through expression profiling or genomic sequencing are either not druggable, or are druggable but there are no approved drugs to target them [20]. With an increasing interest in drug combination screening, the approach discussed here can be readily used as an efficient optimization procedure to identify potential drug targets and their best combinations based on their selectivity profiles and individual responses in given cancer samples.

Several studies have reported the individual expression pattern and function of each hub (TK1, CSNK2B, VIM, YWHAB and HSP90AB1) in different tumors and tumor models *in vitro* [23–31, 42–50]. Here, single siRNA transfections had no detectable effect on the proliferative and invasive properties of cell lines under study. These resutts are not necessarily in contradiction with other studies where inactivation of these hubs had measurable effects, and may be due to different cell lines and culture/assay conditions. That the effect of combination knockdown is larger than that expected from their sum is most likely a consequence of cell signaling pathway redundancy in malignant cells. Consequently, simultaneous inactivation of several hubs may be necessary for successful elimination of potential alternative/crosstalking signaling pathways that are necessary for the maintainance of the maligant phenotype. Our results are supported by the well-known contribution of the individual component of the network targets to cancer development and progression. TK1 promotes cell proliferation, decreases DNA repair efficiency and induces cell death [23, 24, 51]. HSP90AB1 promotes angiogenesis not only as a protein chaperone, but also as an mRNA stabilizer for pro-angiogenic genes, such as *BAZF* [43]. Vimentin is a marker of epithelial-mesenchymal transition (EMT) and promotes invasion and metastasis [25, 52]. YWHAB, a member of the 14-3-3 family of proteins is involved in the activation of tumor/metastasis pathways and inhibition of apoptosis [26, 44, 45, 47]. Similarly, CK-2β promotes EMT and metastasis while inhibiting apoptosis [30, 46, 48, 50]. It is suggested that the concurrent inactivation of multiple hubs is more efficient in the induction of cell death due to the communication failures between functional modules and the lack of alternative pathway options [53]. In agreement with this phenomenon, we observed continued cell death beyond 48 h post siRNA transfection. This observation suggests that despite the short half-life of the top-5 gene mRNA, the transient knockdown was sufficient to significantly affect the stability of a malignant signaling network [54, 55]. Using a modeling approach, Fumiã and Martins [56] showed that while monotherapies were ineffective, drug cocktails were highly effective and necessary for efficient reversal of all hallmarks of cancer. These observations, in association with the results of our previous studies [20, 21], indicate that top-5 genes identified here are potentially valuable druggable targets in invasive breast cancer.

Significantly, the entire set of predicted drug targets identified here has been experimentally validated by available drugs or siRNAs [31, 57–61]. Thus, conceivably, the described approach can be implemented for a substantial number of currently used chemotherapeutic drugs with well-described mechanisms of action as reported in various studies [56]. It is hoped that our strategy will aid in the elucidation of underlying molecular networks of different tumors and histological subtypes. As demonstrated here, this strategy is valuable and can, potentially, add new tools to the armamentarium of drugs at the disposal of oncologists.

The strong differences in the response of MDA-MB-231 and MCF-7 cells to the inhibition of the top-5 targets reinforce the concept of precision medicine, which is considered as a shift away from the *one-size-fits-all* approach. Additionally, we confirmed the notion of categorizing the efficacy likelihood of selected hub targets as high and low according to their connectivity rate (local network entropy) [21], which suggests that precision medicine in terms of targeted therapeutics is a sound new avenue for clinical practice. Indeed, the significant decrease of cell proliferation, migration and invasion, and increased cell death in MDA-MB-231, show that molecular dissection of tumors and multi-target agents should be classified as *high efficacy group treatment* [3], and become a reality in the near future of personalized therapy. Thus, the integration of interactome and transcriptome data allows the effective selection and prioritization of suitable protein targets for drug development and experimental testing or eventual clinical translation among the massive number of possible target combinations.

Compared to conventional cytotoxic drugs that affect both normal and tumoral cells, *synthetic lethality* [62] can address anticancer therapy by optimal hub targeting according to cancer type while sparing normal cells. However, despite the advances in siRNA targeting and compound screening, synthetic lethal interactions between genes and/or drugs have remained extremely difficult to predict on a global scale. Network-based methods provide a convenient platform to find functional interactions enabling the identification of targets and drug combinations for effective and personalized cancer therapies. We were able to induce a maximum of 40% cell death by transiently inactivating top 5 hub proteins in a TNBC cell line, which suggests that increasing the number of deactivated hubs or combining siRNA therapy with drugs may provide synergistic antitumor effects with the current protocol of radiotherapy and adjuvant chemotherapy. According to this rationale, cells sensitized by top-5 target specific drugs (or more) should allow for the reduction of cytotoxic drug concentrations of individual drugs and hence lead to reduced adverse side effects.

## CONCLUSIONS

Based on the analysis of gene expression and signaling protein-protein interaction networks using bioinformatics tools, we tested a combinatorial therapeutic approach, and validated it using cell-based assays. The results clearly demonstrate the effectiveness of this approach to significantly decrease malignant cell proliferation, migration and invasion without any noticeable deleterious side effects to the reference cell line. While these results should be validated *in viv*o using animal models, they clearly support the power of bioinformatics inferences for identification and selection of druggable hubs as well as formulating synergistic combination drug therapies for treatment of breast cancer.

## MATERIALS AND METHODS

### Reagents, cell lines and culture conditions

Unless stated otherwise, all chemical reagents were purchased from Sigma-Aldrich (Oakville, Canada) and tissue culture reagents from Invitrogen (Burlington, Canada), respectively.

MDA-MB-231 (TNBC) cells were cultured in the RPMI supplemented with 10% fetal bovine serum (FBS), 1% L-glutamine, 1% penicillin, streptomycin and kanamycin (PSK). MCF-7 (Luminal A) and the non-tumoral breast epithelial cell line MCF-10A were maintained in MEM media supplemented with 10% FBS and 1% PSK.

### Antibodies

Antibodies, their source and respective dilutions used in this study are listed in Table 1.

**Table 1:** Antibodies and their respectives dilutions in immunoblot assays

Antibodies	Species	Dilution	Source
CSNK2B	Rabbit	1:1000	Thermo Scientific
YWHAB	Rabbit	1:1000	Origene
HSP90AB1	Mouse	1:2000	Origene
Vimentin	Mouse	1:200	Sigma Aldrich
Thymidine Kinase1	Rabbit	1:5000	Origene
Phospho-AKT (Ser473)	Rabbit	1:1000	Cell Signaling
AKT (pan)	Rabbit	1:1000	Cell Signaling
Phospho-c-Raf (Ser259)	Rabbit	1:1000	Cell Signaling
Phospho-GSK-3B (Ser9)	Rabbit	1:1000	Cell Signaling
Phosho-PTEN (Ser380)	Rabbit	1:1000	Cell Signaling
Phospho-PDK1 (Ser241)	Rabbit	1:1000	Cell Signaling
Phospho-AKT (Thr308)	Rabbit	1:1000	Cell Signaling
Phospho-p38 MAPK (Thr180/Tyr182)	Rabbit	1:1000	Cell Signaling
Phospho-p44/42 MAPK (Thr202/Tyr204)	Rabbit	1:1000	Cell Signaling
Phospho-SAPK/JNK (Thr183/Tyr185)	Rabbit	1:1000	Cell Signaling
β-actin	Mouse	1:2000	Sigma
Alexa Fluor 488	Goat	1:2000	Molecular Probes
Alexa Fluor 546	Goat	1:2000	Molecular Probes

### siRNA transfection

Table 2 is a list of target specific (HSP90AB1, CSNK2B, TK1, YWHAB and VIM) and non-specific (scrambled) siRNAs purchased from Sigma-Aldridch (MISSION® pre-designed siRNAs). Transfections were performed using Lipofectamine 2000 (Invitrogen, Carlsbad, CA) and according to the manufacturer's protocol. Briefly, cells were seeded in 6-well plates at 5 × 10^5^ cells/well and incubated for 24 h. Replicate cultures remained untreated (i, control) or incubated with scramble (ii) or individual (iii), or a combination of network targets (iv) siRNAs at 50-100 nM concentration for 6 h. Transfectants were then rinsed with PBS, subcultured and processed for various assays 48 h post siRNA transfection.

**Table 2:** List of the siRNAs used in this study

Gene symbol	siRNA identification	Sequence start (*)
TK1	SASI_Hs_00178715	203
CSNK2B	SASI_Hs_00181021	659
VIM	SASI_Hs_00044033	951
YWHAB	SASI_Hs_00024240	1494
HSP90AB1	SASI_Hs_00055902	1335
-	MISSION® siRNA Universal Negative Control #1	-

(*) Indicates the approximate position where the MISSION siRNA targets the transcript of interest. The displayed nucleotide position is relative to the beginning of the Ref Seq sequence.

### Preparation of cell extracts and immunoblotting

Confluent 6-well culture dishes were washed with cold phosphate-buffered saline (PBS), solubilized in hot SDS sample buffer (10 mM Tris-HCl pH 6.8, 2% (w/v) SDS, 50 mM dithiothreitol (DTT), 2 mM EDTA, 0.5 mM PMSF) and boiled for 10 min. Protein determination was done using Bradford (Pierce) assays according to the manufacturer's instructions. Fifty micrograms of total cellular protein were resolved by SDS-PAGE, transferred to nitrocellulose membranes and processed for immunoblotting. Membranes were blocked for 1 h using 5% (W/V) gelatin in Tris buffered saline-Tween 20 (G/TBS-T). Then, membranes were incubated overnight with primary antibodies (Table 1) in G/TBS-T. After three washes of 10 min in TBS-T buffer, membranes were incubated for 1 h with the secondary antibody (Table 1) and developed by LI-COR IR fluorescent dyes. Immunoblots were performed at least three independent times and a representative experiment is shown in Figures 1 and 7. To evaluate the efficiency of gene knockdown at 24 and 48 h post siRNA transfection, transfected cells were collected to measure the protein level of each targeted gene. Image J was used to quantify the results.

### Cell proliferation and survival assays

For cell growth, replicate cultures were established 48 h after transfection in 24-well plates (Sarstedt, Canada) at 5 × 10^4^ cells/well. At 24, 48, 72 and 96 h after plating, cultures were trypsinized, stained with trypan blue and counted using hemocytometer. The total number of cells/well for each cell line was calculated and plotted for each time point. For further verification, we also assessed cell proliferation by crystal violet staining. Cells were plated in 96-well microtiter plates at 5 × 10^3^ cells/well. At specific time points, cells were washed with PBS, fixed with glutaraldehyde for 10 min, and stained with 0.1% (W/V) crystal violet in 0.2% (V/V) Triton X-100. Microtiter plates were read on a spectrophotometer at 570 nm.

For cell survival, cells were plated in 96-well plate at 5 × 10^3^ cells/well. At 24, 48, 72 and 96 h, 20 μl of 3-(4,5-dimethylthiazol-2-yl)-2,5-diphenyltetrazolium bromide (MTT) was added to each well, and the plates were incubated at 37°C for another 4 h at which time the resulting formazan crystals were solubilized by the addition of 200 μl of MTT solubilization solution. The absorbance at 570 nm was recorded using a microplate reader (Bio Tek Instruments, Winooski, VT, USA). Each experiment for each cell lines was repeated 3-5 times.

### Cell cycle analysis

Six-well plates were inoculated with 1 × 10^6^ cells/well. At 48, 72, 96, 104, 112, and 120 h after plating, cells were trypsinized and washed with PBS and stained with propidium iodide (50 μg/mL in 3.8 mM sodium citrate). Cell cycle analysis was performed using a FACScalibur flow cytometer and CellQuest software (BD Biosciences).

### Focus formation assay

Transfected and non-transfected cells were plated in six-well plates at 5×10^2^ cells/well. Growth media were replaced every 2 days. After 2 weeks, cells were rinsed with PBS, fixed with glutaraldehyde for 10 min, and stained with 0.1% crystal violet. Foci were visualized using an inverted microscope and photographed.

### *In vitro* cell migration and invasion assays

Cell migration was measured by inoculating 5×10^4^ cells in the upper chamber of Cultrex Transwell inserts (polycarbonate membrane, 8 μm pore size) in a 96-well plate (Trevigen, MD, USA). Culture media supplemented with 0.5% or 10% FBS was added to the top and bottom chamber, respectively. Cells were incubated at 37°C and allowed to migrate for 24 h. Migrated cells were detected and quantified using Calcein-AM according to the manufacturer's protocol. The fluorescence of migrated cells was recorded using a 490 nm excitation filter and a 520 nm emission filter using a microplate reader. Matrigel invasion assays were performed according to the manufacturer's protocol (Cultrex Transwell, Trevigen). For each cell line, 5×10^4^ cells were plated in 0.5% FBS media on top of Matrigel-coated invasion chambers (8 μm pore membrane). Media containing 10% FBS was added to the bottom chambers and plates were incubated at 37°C. Membranes were recovered after 24 h incubation and migrated cells were stained with Calcein-AM. Migrated cells on the reverse side of the Transwell membrane were fixed in glutaraldehyde for 10 min, stained with 0.1% crystal violet, viewed under a 20X objective of an inverted microscope and photographed. Each assay was repeated 3 times.

### Soft agar colony formation assay

For this assay, growth media containing 1.2% agarose was used to coat six-well plates. Cells were then trypsinized, and resuspended in 3 ml of growth media containing 0.6% agarose and plated at 5 × 10^2^ cells per well. Cells were allowed to grow for 4 weeks at which time colonies were counted under a phase contrast microscope. This assay was repeated 3 times.

### Statistical analysis

Statistical and graphical data analyses were performed using Prism 5 (Graphpad) software. All quantitative experiments have been repeated using at least three independent times. Quantitative data were analyzed by one-way analysis of variance (ANOVA) and Dunnett's test.

## SUPPLEMENTARY FIGURES


