# Supplementary material for: Validation of a network-based strategy for the optimization of combinatorial target selection in breast cancer therapy: siRNA knockdown of network targets in MDA-MB-231 cells as an in vitro model for inhibition of tumor development
Source: Oncotarget. 2016 Aug 4;7(39):63189–203. doi: 10.18632/oncotarget.11055 (PMC5325356; doi:10.18632/oncotarget.11055)
Supplement: Supplementary file 1 [file oncotarget-07-63189-s001.pdf]

Validation of a network-based strategy for the optimization of combinatorial target selection in breast cancer therapy: siRNA knockdown of network targets in MDA-MB-231 cells as an *in vitro* model for inhibition of tumor development

SUPPLEMENTARY FIGURES

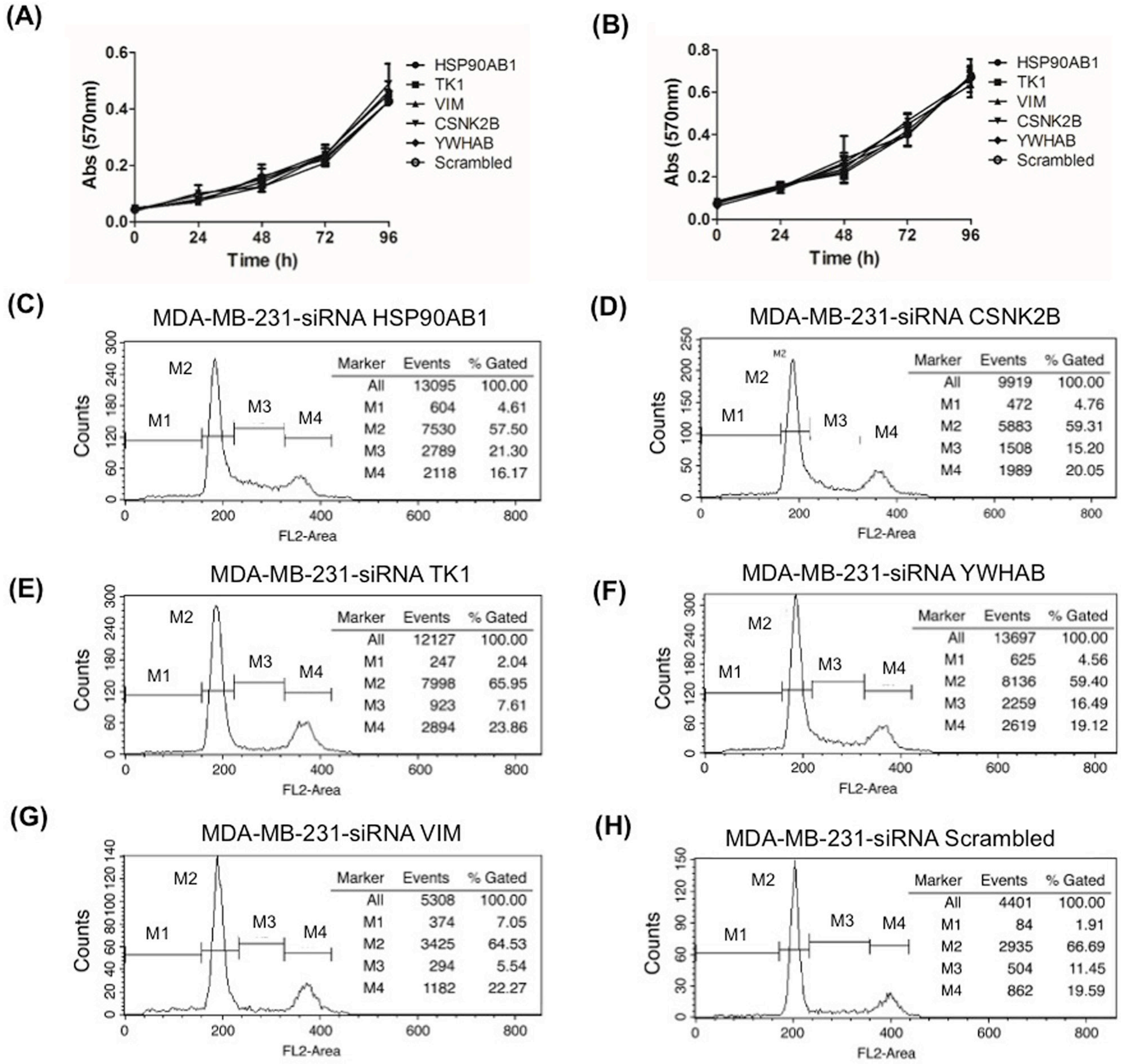

Supplementary Figure S1: The knockdown of individual components of the network target has no detectable effect on MDA-MB-231 cells growth, survival and death. MDA-MB-231 cells were transfected with scrambled siRNA or individual siRNA to HSP90AB1, CSNK2B, TK1, YWHAB and VIM. Cells were processed for growth **A.** and MTT survival **B.** assays as described in Materials and Methods. Percent dead cell in transfectants was determined by propidium iodide staining and cytometry **C-H.** The percentages of cell death (sub-G1) are indicated as “M1”.

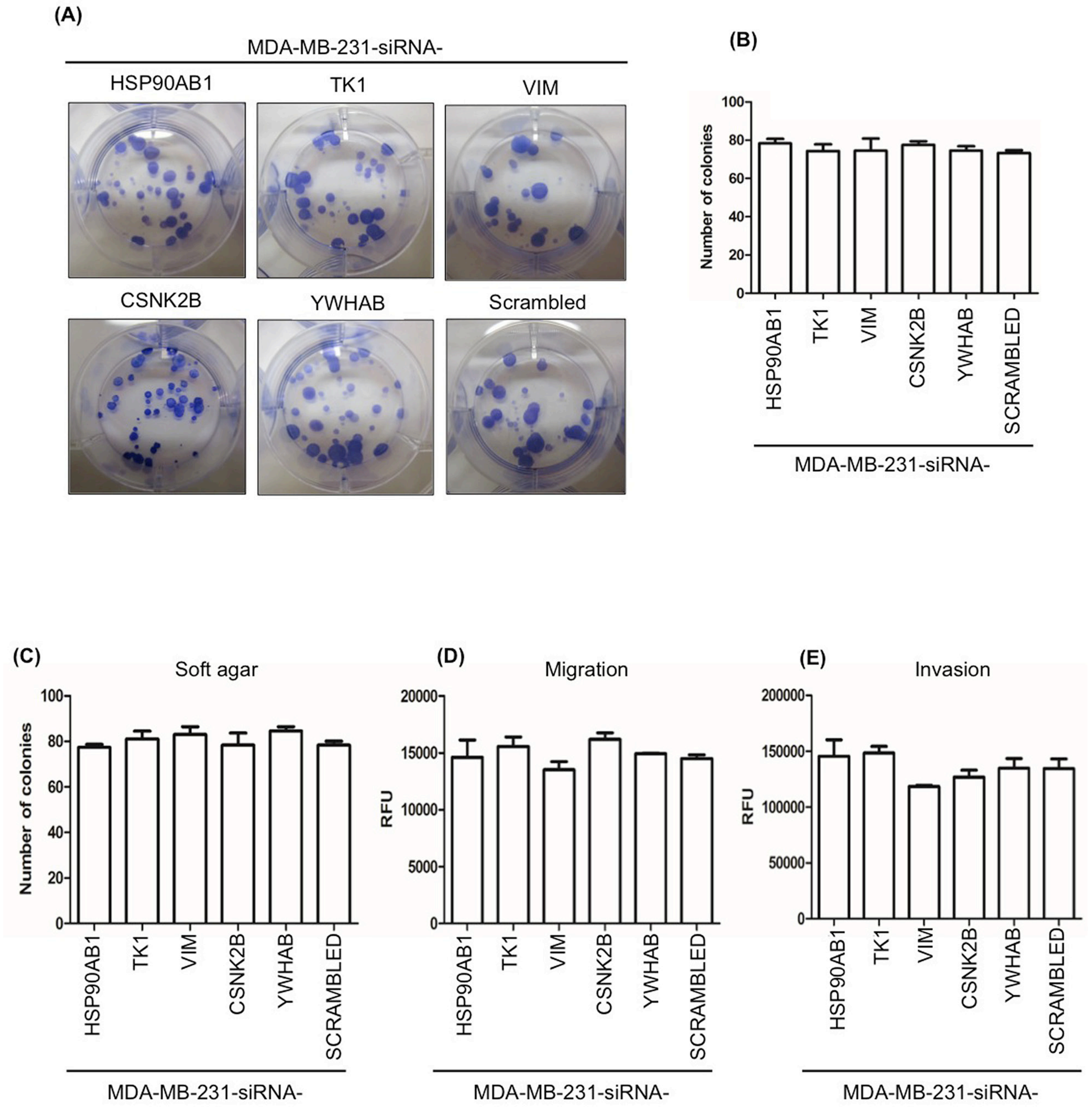

Supplementary Figure S2: The knockdown of individual components of the network target has no detectable effect on MDA-MB-231 cells foci formation, growth in soft agar, migration or invasion. MDA-MB-231 cells were transfected with scrambled siRNA or individual siRNA to HSP90AB1, CSNK2B, TK1, YWHAB and VIM. Cultures were processed for foci formation **A,B.**, soft agar growth **C.**, migration **D.** and invasion **E.** as described in Materials and Methods. Histograms represent the average number of colonies, migrated/invaded cells ± SD of 3 independent experiments.
